# To Be or Not to Be a Female Gamer: A Qualitative Exploration of Female Gamer Identity

**DOI:** 10.3390/ijerph19031169

**Published:** 2022-01-21

**Authors:** Daria J. Kuss, Anne Marie Kristensen, A. Jess Williams, Olatz Lopez-Fernandez

**Affiliations:** 1 International Gaming Research Unit, Cyberpsychology Research Group, Department of Psychology, Nottingham Trent University, Nottingham NG1 4FQ, UK; 2 Center for Visual Cognition, Department of Psychology, University of Copenhagen, 1165 Copenhagen, Denmark; amhk@psy.ku.dk; 3 School of Psychology, Institute for Mental Health, University of Birmingham, Birmingham B15 2SQ, UK; a.williams.10@pgr.bham.ac.uk; 4 Foundation Health Research Institute, Fundación Jiménez Díaz University Hospital, 28040 Madrid, Spain

**Keywords:** female gaming, qualitative analysis, gaming culture, game studies, female gender, identity

## Abstract

The literature on online gaming has generally focused on male gamers and has been dominated by negative aspects of gaming. The present study addresses the gender gap in this field by exploring experiences of female gamers further by unravelling several positive experiences alongside some potentially harmful tendencies connected to gaming, including female gamers’ wishes and ambitions for their future gaming. A total of 20 female adult gamers across Europe were interviewed and results were analysed using thematic analysis. Four main themes were identified: (i) to be or not to be a (female) gamer; (ii) improving social skills and levelling up on mental health; (iii) not always a healthy escape; and (iv) there is more to explore. The present study is one of few empirical studies regarding the construction of self-image, and experiences of female gamers. It has showed participants have a history as gamers from adolescence, but still face problems derived from the stigmatised internal gender self-image. Externally, female gamer stigmatisation may result in sexism, gender violence, harassment, and objectification. Additionally, females may decide against identifying as gamers, engaging in social gaming interaction, or hold back from online gaming in general, thereby missing out on the opportunities for recreation as well as social and psychological benefits that gaming brings. There is, therefore, urgent need for more research and actions to promote change, equity, education, and security for female gamers as well as their male counterparts. Game developers would benefit from understanding this large gamer demographic better and tailoring games for women specifically.

## 1. Introduction

Traditionally, the gamer stereotype has been that of an unpopular, overweight, and socially inept male gamer [1]. Recent statistics, however, indicate that this stereotype is far from accurate. Of American video game players, 45% are female (Entertainment Software Association (ESA) [2]). According to the International Software Federation of Europe (ISFE [3]), 47% of European video gamers are women, with an average age of 32 years, and they represent 53% of mobile and tablet gamers, a higher proportion than men. At the same time, video games tend to be designed by men for men, and female needs and requirements are not sufficiently taken into consideration by game designers [4]. Moreover, women are a marginalised and poorly understood population when it comes to gaming [4]. Game developers would benefit from understanding this large gamer demographic better and tailoring games for them specifically.

The not-for-profit organisation Women in Games looks for parity and equity for females in the gaming industry and esports. According to them, the gaming industry is “the most gender imbalanced of all the creative industries” due to women being underrepresented as executive managers of games companies, and only 5% of competitive e-gamers are women (Women in Games [5]). As highlighted by the BBC [6], women are rarely successful in high profile esports/gaming championships, for example Dota 2. This reference also illustrates that the minority status of female professional gamers has received increased media attention outside of academia in recent years. There are a few exceptions to the rule, and a small number of female gamers and female game designers are increasingly appearing in the spotlight, including Brenda Romero (Dungeons & Dragons: Heroes) and Jane McGonigal (SuperBetter).

Using data derived straight from videogames through online videogame databases, research has indicated that female avatars are overly sexualised and often take on secondary roles within the game [7]. Female game characters are often represented in a hypersexualised way with large breasts (i.e., disproportionate to body size, exposed skin, accentuated by clothing) and bottoms (with exposed skin or adornments) and small waists (bare skin, exaggerated waist-to-hip ratio), as well as overly sexualised movement (e.g., needless undulation and jiggling [7]).

### Theoretical Background

Consequently, the present-day gaming culture can be considered misogynistic and immature [8]. Of concern is the fact that not only are women being objectified in-game, but this objectification can also translate into settings outside of the game, such as receiving sexualized comments (FG11) or unwanted photos of male genitals (FG5). Research shows that men who play videogames that have representations of hypersexualised female characters tend to have sexist attitudes towards women outside of games [9], and these men are even more likely to accept rape myths [10].

There is stigma attached to being a female gamer, and women are often objectified and harassed in videogames. The stigma attached to being a female gamer has measurable negative impacts on women. Being considered a ‘bad’ gamer has led to poorer performance in female gamers in experimental Implicit Association Tests [11], and they are perceived as less competent gamers than their male counterparts [12]. 

There is some evidence to suggest that women have different motivations for gaming in comparison with men. Female USA mobile gamers play to combat boredom and for enjoyment, to be autonomous, and to create relationships [13]. Men, on the other hand, play for success and competition [14]. In addition to this, women play for competition, recreation, and social reasons. For some women, recreational and social gaming motivations are associated with internet gaming disorder (IGD), whereas in men, social gaming motivations are associated with lower IGD [15]. IGD was first included in the American Psychiatric Association’s (APA) most recent diagnostic manual, the *DSM-5* [16], as a condition requiring further research to be included in the main manual. More recently, gaming disorder (GD) has been included in the eleventh edition of the World Health Organisation’s (WHO) diagnostic manual, the *International Classification of Diseases* (*ICD-11* [17]), suggesting that for some excessive gamers, their activities become problematic to the extent that they may require professional help [18].

Rather than being a potentially problematic pastime activity, gaming can have a number of positive outcomes for gamers. Research indicates that it can have therapeutic functions, both physiologically as well as psychologically, and can increase gamers’ wellbeing, life satisfaction, and social support [19]. Moreover, the ISFE [3] suggests that gaming can lead to beneficial outcomes in females. They indicate that the probability of pursuing a career in Science, Technology, Engineering, and Mathematics (STEM) is increased threefold in girls who play videogames in comparison with those who do not. This, however, also works the other way round, as noted by Williams [20]: “girls who do not play become women who do not use computing technology (…) and certainly do not aspire to make games” (p. 16).

Taken together, the literature on online gaming has generally focused mainly on male gamers [21] and has been dominated by negative aspects of gaming. The present study addresses the gender gap in this field of research by exploring experiences of female gamers further by unravelling several positive experiences alongside some potentially harmful tendencies connected to gaming, as well as female gamers’ wishes and ambitions for their future gaming. The issues and effect on women related to male perceptions of female gamers, portrayal of female game avatars available, and interaction with the online community are investigated further in an upcoming project. The objective of this qualitative study was to explore in depth what it means to be a female gamer for women who regularly play videogames.

## 2. Materials and Methods

### 2.1. Design

The current study was a qualitative study using semi-structured interviews. These considered the perceptions and experiences of video gaming as a female. The Nottingham Trent University Ethical Review board approved the current study as part of a wider project (Female Gamer) wherein the goal was to gain an in-depth understanding of female video gaming in community samples. 

### 2.2. Participants

Participants were 20 women, ranging in age from 21 to 39 years (*M* = 26.06, *SD* = 4.63), from a variety of countries. Eighty percent (*n* = 16) were from Europe, primarily the UK (*n* = 14), Spain (*n* = 1), and Norway (*n* = 1). The remaining participants were from the USA (*n* = 3) and Canada (*n* = 1). The majority of women were employed (90%; *n* = 18), and 40% (*n* = 8) worked in research in some capacity. Fifty percent of participants selected to take part in a written interview, with remaining participants evenly split between audio or in-person interviews. More information is provided in Table 1.

The participants who volunteered for the interviews responded to a recruitment advertisement for “Female Gamers”. On this basis, we will categorise and refer to them as such. In this paper, the participants are not referred to as women, as they have not explicitly identified as such. 

### 2.3. Procedure

The sample were recruited following the completion of a previous study [22]. Interested individuals were encouraged to leave contact information at the end of the related survey. All participants who left contact information were then contacted with an invitation to interview. Participants were offered a GBP 20 voucher as thanks for their time. From this, 20% wished to engage. To take part in both studies, participants needed to play video games, be able to communicate in English, and identify as a woman.

Informed consent was obtained, with indication of how the individual wished to be interviewed and their availability. A variety of interview methods (in-person, Skype audio, or Skype written) were offered for convenience, e.g., a written Skype interview if participants had limited access to microphone or sound equipment or if they were nervous about speaking out loud. All interviews were conducted privately, with the participants’ permission to record the session. Private interviews encouraged more in-depth and personalised responses.

Twenty semi-structured interviews were conducted throughout June 2018. Each interview was guided by 34 open-ended questions developed by two of the authors (O.L.F. and D.J.K.; which are available as an online supplement—see Appendix A). These covered the experiences and impact of gaming and views on specific female-related topics within video games and gaming culture. All interviews were conducted by one of the authors (A.J.W.). Spoken interviews (Skype audio or face-to-face) took approximately 60 min, whereas the written interviews ranged from 90–210 min. At the end of each interview, the interviewer asked participants if they had any additional questions or comments relating to female gaming. 

### 2.4. Analysis

As all interviews and transcription was completed by A.J.W., the researcher was immersed in the data from collection. This allowed for initial identification of coding to begin and ensured data saturation was achieved before closing recruitment [23]. All authors are females and casual gamers, allowing them to analyse and interpret the data from within a female gaming perspective. Following full anonymization of all interviews, thematic analysis was utilised to analyse the data. This process followed the guidelines offered by Braun and Clarke [24]. After reading and familiarising with the data, semantic codes were used to build independent frameworks (A.M.K., A.J.W.). Following comparison, recurrent patterns were clustered into larger themes and subthemes within them.

In order to secure internal homogeneity and external heterogeneity, themes were collapsed and rearranged during the reviewing phase among the research team [25]. This was to secure internal consistency and that themes were distinctly different [25]. Upon this phase, the data were recoded systematically to ascertain that the full dataset was embraced by the themes in use, and themes were refined alongside. Theoretically, we were interested in the experiences and insight of the 20 participants. Therefore, their statements are central to our analysis and are explored and exemplified further through the existing literature.

The codes used to refer to the participants were: ‘FG’ for female gamer and a number as identification number (ID), i.e., FG1 is the first female gamer interviewed. The codes were used to refer participants’ quotes, adding between parenthesis their respective participant’s age in numbers and the professional status as main variables which describe characteristics of the participants in relation to the meaning provided in an analytic theme or subtheme (see Table 1).

## 3. Results and Discussion

**Figure 1:**
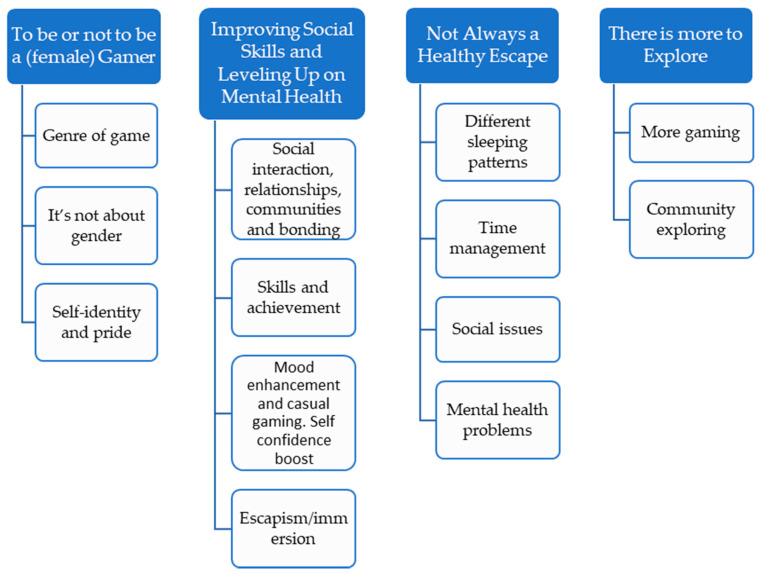
Overview of identified themes and subthemes.

Four major themes were identified: (i) to be or not to be a (female) gamer; (ii) improving social skills and levelling up on mental health; (iii) not always a healthy escape; and (iv) there is more to explore. Each theme is presented in Figure 1, with associated subthemes. Findings of the present analysis revealed participants viewed gaming as a nuanced and multifaceted activity, in which an array of psychological and practical factors influence the effect it has on its players and their surroundings. For our participants, gaming serves various purposes, which we will exemplify and elaborate on through quotes in the following sections.

### 3.1. To Be or Not to Be a (Female) Gamer

The first theme considers issues associated with identity and gender in relation to gaming. In the literature as well as by our participants, self-image and female gamer identity were perceived in relation to the typical gamer stereotype. This will be addressed in relation to this theme. The typical gamer stereotype has been presented as a pale geeky teenage boy hiding in his basement, playing massively multiplayer online role-playing games (MMOPRGs) or first-person shooter games (FPS), spending the majority of his time online rather than offline [4,26].

Some of the participants identified as being a “real” gamer, as they spend a substantial amount of time gaming. Similarly, several participants mentioned that they see themselves more as females who game than “female gamers” as a category (FG1, FG6, FG8, FG10). For instance, when considering her gaming, participant FG1 (33, Researcher) said that “*I don’t really think of it as a woman. I think of it as an individual I suppose*”. Here, the participant refers to the label of “gamer” as “it”. This highlights that the participant does not feel the need to gender the label “gamer” as male or female. Participants generally agreed that they did not perceive their gaming as an activity related to their gender.

Although participants took part in this study due to being “female gamers”, only some of them self-identified with the label of “gamer”, and even fewer specifically as “female gamers”. The perception of whether the women identified as gamers or not ranged from one extreme to the other, from: *“Gamer is probably my primary identity. I will always be a gamer, and I only want to make friends with and date other gamers”* (FG8, 28, unemployed) to “*I guess I wouldn’t call myself, although I do gaming, I wouldn’t call myself a gamer in the same sense that I don’t call myself a cross-stitcher, I guess the hobby does not identify me, kind of thing”* (FG5, 28, unemployed).

These statements highlight the diversity amongst participants, suggesting that gaming is used for different reasons and gaming has different meanings among participants. The excerpts emphasise that gaming can vary from being a core characteristic of one’s identity to being detached from one’s self-identity and more of a casual hobby (FG12, FG13, FG14). One participant expressed that she fully embraces the gamer aspect of her identity in spite of some of its negative connotations to people around her:

*I’M A GEEK AND PROUD OF IT!! Gaming is important in my life, both tabletop, and starting in a larger degree digital. I am not afraid to tell people I am a gamer, and I show off my geekiness through t-shirts of all kinds with geeky writings. For me ‘Geek’ is not a negative term, even though some, like my mother, tries to make it so. I wear that label as a show of pride.*

(FG7, 39, preschool teacher)

In this quote, the pride and importance related to gaming stands out clearly. The word ‘geek’ is turned on its head, as the participant is confident in expressing that she is a gamer. This change of discourse is supported in the study by Paaßen et al. [4], according to whom playing popular games can now be considered ‘cool’ rather than as a “nerd or geek practise” (p. 430). The change of discourse was recognised amongst participants in relation to their online gaming. *“[Gaming] was for ‘geeks’ but geeks weren’t cool at all, not like now with ‘geek chic’”* (FG4, 35, radiographer). “Geek chic” mirrors the experience stated above by participant FG7. However, FG4 recognised the former stereotypical perception of gamers was negative and no longer felt affected by this:

*I used to be embarrassed to be a gamer. Many of my school/university friends don’t know. As I’ve gotten older I don’t waste time being embarrassed about something I enjoy. I’m not sure if younger women would still say that—as a gamer when it was only for “geeks” I didn’t want to be known as a geek so pretended I didn’t play.*

(FG4, 35, radiographer)

Participants discussed the effect of gaming-related stigma and the geekiness of being a gamer. Gaming-related stigma could cause some to act as if they do not play. When female gamers hide the fact that they game, the result is that other female gamers risk feeling more like the odd ones out. The gamer stereotype also seemed to affect other participants, who distanced themselves from the label of being a “real gamer” on the suggestions that “real” gamers play every day (FG13), are heavily invested in their games, and are part of online communities (FG14), as well as playing specific types of games, such as MMOPRGs (FG2, FG12, FG16); *“like I wouldn’t call myself a gamer even if I play The Sims 30 h a week”* (FG16, 23, lab technician). Although female gamers spend approximately as much time on gaming as their male counterparts [4,20], several participants expressed that gaming is still primarily perceived a male activity by the society and community around them (FG5, FG14, FG16, FG18).

Assumptions about gender and gaming are also cultivated by female gamers, even with current information regarding the gaming industry showing that both genders are half of the present markets since 2018 [27,28]. Although most people have moved on from the image of the isolated and pale-skinned teenager according to Paaßen et al. [4], gamers are still associated with being male:

*And the more people I came across growing up who thought that either it was silly that I played because I was a girl, or that I was no good at them because I was a girl, or oh I must have meant some silly little girlie game because I was a girl.*

(FG1, 33, Researcher)

The above quote demonstrates that female gamers are often judged based on gender rather than abilities, activities, or accomplishments. FG18 (PhD Student) identified as a female gamer and considered her experience different from the male gaming experience by expressing that *“I think the whole experience from pre-buying to playing is different for men and women.”* As the quote exemplifies, participants frequently met a stereotype of female gamers playing ‘silly’ games and were not taken seriously. Similarly, the first female winner of a Magic Card Grand Prix, Jessica Estephan, mentioned in an interview with Forbes: “*The thing is, when women my age grew up, we weren’t really allowed to like “boy things.” So if I started through my own independent will at 19, but my male counterparts have been playing and thinking about games since they were young, there’s a significant disadvantage there*” [29]. This quote is a good example of how discourses of male and female activities in childhood affect future skills and experience and contribute to keeping stereotypes and discourses alive [4]. Childhood activities discussed by participants who experienced negative responses from others related to gameplay and gender. Consequently, they caused some gamers to question their continuation of gaming. One strategy that participants used to avoid unwanted attention was by using male avatars or non-female pseudonyms (FG10). This was done to avoid unwanted personal interest and to avoid being given ‘special treatment’: *“(…) I just want to play my game, I don’t want answer questions about my relationship status or erm, what I’m doing today or you know”* (FG6, 24, Influencer and Sponsorship Manager). This quote suggests that more gender equity is still needed within this area. Similar issues were mentioned by other participants: *“(…) as soon as you put a picture of a young woman you get all these people hounding you for like sexy message kind of thing. (…) If I don’t feel safe playing it then I won’t play it”* (FG5, 28, unemployed). This quote exemplifies that in gaming cultures, harassment and unwanted attention might lead some women away from certain games. Women are just as likely to play online games as men, and it is just as justifiable for females to identify with being a gamer. Increasing equality across genders and ages in gaming is vital [21].

Coping strategies implemented by female gamers such as hiding their identity or avoiding verbal communication have also emerged in other studies [8,30] to cope with online harassment. A large questionnaire study presented by Lenhart [31] found that 71% of teenage boys used a voice connection when gaming in comparison with 28% of girls playing in networked environments. According to Vella et al. [32], gamers who express their ‘true selves’ via their avatars as opposed to using anonymous written communication are more likely to form long-lasting social connections. On this basis, design changes need to be implemented to aid female gamers in using voice connections and not having to protect themselves by hiding behind alter egos in the form of their gaming avatars. 

In conclusion, the female gamers that we interviewed generally expressed that they preferred to identify as gamers without using the gendered category. Several participants mentioned that gender did not play a role in their perception of their own gaming. When playing, they often received personal questions and special treatment if they were identified or appeared as female. The issues of harassment and inequality in gaming are known from the literature on gender issues in gaming. It is important to spread the information that women are just as likely to game as men. The typical gamer is a lot more diverse than most people used to think [4].

### 3.2. Improving Social Skills and Levelling Up on Mental Health

This theme explores how and what participants discussed as social, relational, and mental gains in relation to their gaming. The analysis revealed a focus on the skills and achievements that the participants acquired with a sense of pride, and the social bonds that are enabled and strengthened through gaming. Mental health was discussed in common issues such as stress, depression, anxiety, and problematic gaming. This theme also presents the benefits of gaming on such issues.

Including the diagnosis of IGD into the diagnostic manuals of the *DSM-5* [16] and GD in the *International Statistical Classification of Diseases and Related Health Problems* (*ICD-11* [17]) adds the risk of overpathologising everyday behaviour [33,34]. There appears to be an exaggerated focus on the negative consequences rather than the benefits of gaming in the literature and the public news media. However, during the interviews, the positive utility of gaming was a central theme for our participants and will be elaborated further in relation to the existing literature; *“(…) certainly from maybe older generations there’s a bit of a stigma that’s it’s not good for you but it’s like why isn’t it? You know it’s just [a] medium like anything else, it’s like storytelling.”* (FG2, 27, PhD student). This excerpt demonstrates the attitude that gaming has received an unnecessarily bad reputation in public discourses.

Several participants mentioned how gaming strengthened their social abilities and connections in the offline world as well as online. Some participants found games particularly helpful regarding practising and learning about social interaction.

*It brings people together because you’ve got a common, shared interest erm, it’s good banter, it’s good stress relief, and honestly you know for a lot people out there that just don’t have much or just aren’t very happy in life, video games can provide a very escape for them. (…) I don’t think gaming deserves the hate it gets from the parents out there.*

(FG6, 24, Influencer and Sponsorship Manager)

The beneficial elements of gaming highlighted here are that games are typically fun, and in this participant’s perception, parents tend to overlook the positive aspects related to gaming. Additionally, games were discussed as providing an escape for people who lack resources and opportunities in everyday life. The stereotype of the online gamer might often be associated with solitude and loneliness [4]. Here, however, participants felt that games can be a way of bringing people together. In the case of female gamers, the social function of gaming appears as a protective factor safeguarding against developing mental health problems. Other participants highlighted social gaming aspects, such as spending time and bonding with family members, despite lacking many common interests. Previous studies report that women are often introduced to gaming by male family members, friends, or a partner [4], supporting our findings.

Games were mentioned to enable the establishment of new social connections and maintenance of existing relations both offline (FG9, FG10) and online (FG10, FG16). Gaming was also described as enhancing skills when dealing with serious problems and linked to language acquisition:

*(…) so I think the positive consequences of you know being a gamer and growing up with video games, you know it helped me master the English language far beyond what school could teach me. Helped me speak English better, it helped me understand life better, I mean those games sometimes deal with very serious issues and erm, if you’re playing online then you learn social skills, you learn how to interact with people, because it’s so much easier online than it is in person.*

(FG6, 24, Influencer and Sponsorship Manager)

This quote indicates not only several benefits associated with gaming itself but also the challenges and rewards associated with gaming with other people. It was discussed that the virtual world may function as a steppingstone for practising social interaction, as offline social interaction may be perceived as easier than establishing offline connections, which has been shown in previous research on problematic gamers and problematic Internet users [18,35]. This might be because there is less responsibility and there are fewer expectations regarding people that one might not have to ever meet in an offline situation, compared with classmates and family with whom one interacts regularly. Socially, online gaming offers the unique opportunity of hanging out with people who are completely dissociated from one’s everyday contexts; *“I wanted to hang out with my online friends who didn’t know me irl [in real life] so I could be whatever I wanted rather than myself”* (FG4, 35, Radiographer). This quote suggests the freedom of being able to create an avatar or an alter ego who is not constrained by real-life circles of friends and work life and for whom impression management is important. The user–avatar relationship has been highlighted in previous work, indicating the importance of the avatar for the user in terms of self-presentation in an idealised form, approximation of the real self to the idealised self in an offline context, and overcoming offline boundaries and deficiencies [36].

Regarding benefits of gaming, several participants noted that aspects such as engaging narratives and aesthetic virtual designs might appeal more to female than male gamers in comparison with violent FPS games, which several of the participants found relatively uninteresting. Thus, the aesthetics of some game genres can prompt female gamers to play them.

*But when I think male gaming I just think of games with a lot of bullets and explosions and things on fire and if I’m thinking female gaming I’m thinking something sort of more, erm, brainy or creative or more sort of wordy interactions sort of valuing more interactions between players than sort of you have to shoot A, B and C, kind of thing.*

(FG5, 28, Unemployed)

This is one amongst several examples of gaming being an enriching and stimulating activity that is enjoyed and valued. Participants in our study explicitly appreciated acquiring new skills and achieving new in-game milestones. Whilst some participants highlighted game-related skill acquisition (FG19), others explained that they gained new skills transferable to other aspects of life:

*Positively, I think gaming has developed my brain by improving my reaction time and providing me with knowledge, such as teaching me grammar at a young age and how to deal with problems. It also taught me some common sense and how to talk to others, even though it sometimes does not translate well to reality.*

(FG17, 21, donut vendor)

Similar to some of the improvements in social abilities mentioned earlier, this quote reveals how to some gamers the benefit of playing extends beyond the game itself. Studies have found gaming increases focus and attention, develops cognitive and non-verbal skills [19,37]), and improves well-being and relieves stress [38]. According to some participants, games lifted their mood by raising a feeling of pride in themselves when others witnessed an achievement. Moreover, studies have found that games can enhance the feeling of competence through incremental challenges and opportunities for positive feedback [38,39].

Although some participants found enjoyment in acquiring skills and seeking challenges, others enjoyed the opposite, explicitly playing for enjoyment rather than achievement (FG15, FG18). Playing games more casually than competitively appeared to be associated with gender according to some participants. Previously, women were found to play more casual and less time-demanding games because they had fewer and smaller chunks of leisure time than the male respondents [37]). Casual gaming may be related to the mindset with which gaming is approached:

*I think that I would be more likely to like say that I’m a casual gamer sometimes more than basically any man. I think that erm, you know how it is, it’s like they want to be the best and like all that stuff and I think that I play more for fun.*

(FG10, 25, Software Engineer)

This quote represents how female gamers often look for different things in video games [8]. As female gamers are often overlooked as a user group and perceived as incongruent with the typical gamer identity [4], they might have a lot to gain by becoming more visible as a group to gaming communities, the gaming industry, and society as a whole.

Some subthemes which emerged throughout this section mirror the three overarching gaming motivations presented by Yee [40]: immersion, social interaction, and achievement. These describe the different reasons why people game. Winning is generally not top priority to our participants, and overall they attributed competitiveness more to male gamers. This finding is in agreement with Yee’s study [40], in which he found males scored significantly higher on achievement, whereas females rated themselves higher on the social component. These findings were also partly explained by age. The interest, in using gaming to maintain social relationships, was also pointed to as one of the ways in which females differ from their male gaming counterparts [8].

As addressed previously [41], researchers and popular media share a common responsibility not to cherry-pick and enlarge the sensationalist and negative stories associated with problematic gaming. Recently, attention on IGD has increased as part of its inclusion in the research appendix of the *DSM-5* in 2013 [16]. The gaming motivation immersion [40] appeared in this analysis, as our participants expressed that gaming offered an opportunity to let themselves immerse in a parallel universe.

This temporary break from reality can be a useful way of distraction from rumination and potentially harmful thoughts (FG5). It also offered chances to play with other personality characteristics and personas than one’s everyday self: *“(…) if I’ve had a bad day I might want to be more immersed so that’s when I would probably turn on a strategy game.”* (FG1, 33, Researcher). As strategy games are more demanding, immersive, and time consuming than more casual games [37], the participant chooses this type of game to escape the offline world mentally for a while. Here, gaming works as a welcome distraction. Gaming can act as a way of “*switching off*” (FG1, 33, Researcher) and as “*checking out for a bit*” (FG12, 25, PhD student). Both are examples of the stress relief gaming can offer.

Games offer a distraction from everyday life but also a place in which one can build skills and abilities. In this way, several participants presented games as a safe context for practising social skills and building confidence. They can be a place for feeling good at something (FG17) and seeing one’s progression clearly relative to ongoing goals: “*I think it’s quite nice when there are little goals that you can achieve, like during PhD you don’t really get that. So it’s nice to have them short term goals and feel that you’ve achieved something while you’re playing*” (FG12, 25, PhD Student). Accomplishments are regular, achievable, and immediately rewarding in video games [38]. In contrast, when working in academia, milestones and rewards demand long-term persistent efforts, if they are achieved at all. Studies have described academia as an arena characterised by repeated rejections in many forms (e.g., funding proposals), as well as the sense of failure associated with impostor syndrome and burnout [42]. These issues are not just connected to academia but are prominent in other occupations, such as amongst health professionals [43] and teachers [44]. In comparison with these professions, online gaming allows for self-improvement by advancing to new levels, learning new skills, and perhaps advancing into more complex and challenging games.

Perceiving gaming from this point of view, it may serve as a protective factor regarding mental illness. Gaming can act as a coping mechanism and a way of handling difficult emotions through distraction, but it also highlights the complexity of this field and the multiplicity of gaming motivations, which can be linked to positive mental health outcomes [38].

In short, gaming appeared to improve the participants’ moods in multiple ways. It provided engaging entertainment and allowed participants to let themselves immerse in stimulating narratives, as several of the participants highlighted that they enjoyed the storylines as well as the aesthetic content of the games. Gaming was also perceived as an outlet and escape route from difficult situations and mental states. It offered a functional coping mechanism. To several participants, gaming involved joyful social activities online as well as offline, bringing together existing friendships or new acquaintances met online. To some, the virtual world offered a place to practice social interaction with fewer perceived risks than the offline world and skills and achievements that might appear more tangible and within reach than in other parts of their lives.

### 3.3. Not Always a Healthy Escape

According to Lopez-Fernandez, Williams, Griffiths, and Kuss [8], problematic and potentially harmful gaming in women is rarely addressed in the literature. This is one of the reasons why this is an important area to explore, especially as gaming addiction often goes unnoticed in females according to clinicians [45]. Although several participants felt enjoyment and detachment from responsibility during the immersion experienced while gaming, other participants expressed that sometimes losing themselves in the virtual world had consequences in the offline world:

*Also it has an impact on the fact that I need to take care of my house, and I find myself forgetting time when I am playing video games. Sometimes I find myself longing for my computer so I can continue to play.*

(FG7, 39, preschool teacher)

This is one amongst other examples (FG12, FG16) where gaming takes time away from offline life activities, causing problems in everyday life. This statement suggests the presence of salience, withdrawal symptoms, and mood modification—symptoms of GD [16,46]. Further potential harms and consequences are discussed in the following section.

Some participants discussed that excessive gaming has sometimes proved detrimental to their mental health:

*I have started hallucinating a bit after having played certain games for too much. At times I find myself losing focus in real life when thoughts of my gaming grabs me, even though sometimes my gaming has strengthened my ability to focus in real life. And also I believe I find myself sometimes so consumed in gaming that I forget things outside the digital world that I also should focus on.*

(FG7, 39, Preschool teacher)

This quote evidences the complexity and ambivalence associated with gaming that shows throughout these interviews. Gaming plays a part in both strengthening and compromising focus in offline life. Therefore, categorising it as mainly adding or subtracting from everyday life can be difficult. The quote seems like an example of game transfer phenomena (GTP), which according to Ortiz de Gortari and Griffiths [47] is often associated with “*having a pre-existing medical condition, playing 3–6 h sessions at a time, and playing for immersion, exploration, customization, mechanics and escape from the real world (…)”* (p. 195). GTP is common and involves altered perception, involuntary thoughts, and behaviours such as pseudo-hallucinatory experiences after playing video games [47]. A recent study found that gamers with poor self-concept clarity were more likely to let themselves be absorbed in the virtual world, increasing the risk of gaming addiction. This may be due to compensating for lack of offline-world identity [48].

Several participants expressed that their gaming habits were hardest to control during their teenage years (FG10, FG11). One participant discussed gaming interfering with social relationships and mood: *“(…) I think gaming has become a crutch for my mood and have probably been addicted in the past. At times I would choose gaming over my relationships with people”* (FG17, 21, donut vendor). Gaming becomes problematic when it harms the individual’s functioning in everyday life and the ability to engage in normal interaction and duties [41]. The fact that gaming can be addictive [49,50] might be explained by a range of aspects and mechanisms. These include the use of unpredictable reward designs such as nudging in-game purchases in a ‘freemium’ model that blurs the lines between gaming and gambling by incorporating variable-ratio reward schedules [51].

Some participants found themselves immersed in specific games, and perceived that new games in particular are difficult to discontinue:

*In terms of not being able to put a game down my favourite series has always been Pokémon so when a new game comes out in the series I end up playing it through the night which can disrupt my schedule.*

(FG11, 24, data curator)

This participant found that gaming interferes with sleeping patterns. Several studies have found that gaming can cause a dissociative state, in which gamers experience a sense of disembodiment and lose their sense of time and place [52,53], corroborated by individuals in treatment for their addictive gaming [54]. This sense of detachment from the offline world might both cause gaming to become excessive, as gamers may forget about offline world responsibilities, but also because it can function as escape from social and mental problems [55]. Moreover, GD has been found to be associated with several mental health issues, and in this realm, causation is unclear [49,50]. However, it must be emphasised that meeting the WHO’s [17] GD diagnostic criteria implies severe dysfunction obstructing leading a normal everyday life.

Relatedly, the findings by Yee [40] show that the online gaming motivation immersion is the strongest predictor of problematic usage, indicating that it might be an important factor to monitor, e.g., when screening. However, the immersion motivation is also strongest connected to both procedural and declarative knowledge acquisition [56]. The gaming motivation of immersion appears as double-edged sword. It offers relief from everyday problems and aids learning but can also get out of hand and result in problematic gaming. Participants used gaming as an escape from the offline world, indicating that escapism was predictive of addictive MMORPGs playing [57]: “*Because at the end of the day, video games aren’t life [laugh] it’s not really life, erm, so I think it’s better to go out there and experience actual life, than a simulation of that*”(FG3, 29, PhD student). However, desire to game less was not a common pattern. While other participants found that gaming sometimes interfered with their everyday life in a problematic manner, they did not desire to reduce their gaming. Furthermore, participants stated that online games are a safe space for training offline world skills. However, the participant cited above highlighted that practicing them in the offline world is completely different.

In conclusion, female gaming offers ways to escape the offline world, including its social demands and responsibilities. It might provide temporary relief and distraction from conflicts or uncomfortable circumstances, but prolonged withdrawal can make the physical reality even more overwhelming. This can result in preferring gaming to social relationships and academic work, as was the case for some of our participants [58].

### 3.4. There Is More to Explore in Gaming and Online Communities

This theme considers female gamers’ future ambitions and intentions. Based on the stereotypes presented earlier, one might not expect female gamers to perceive it appropriate for themselves to pursue gaming and its communities further [4,22]. However, the extent to which this may be the case is unravelled further in this section.

In relation to their gaming, participants generally expressed a wish to keep things exactly as they were (FG4, FG6, FG10). A small number wanted to game less (FG3), and the majority preferred to game more rather than less. Time was the main constraining factor (FG11).

*I would like to explore the digital gaming world, to find maybe some multiplayer gaming communities that I can feel comfortable in, with a theme that appeals to my seeking of a relaxed, nice time. And as we speak I am already starting to think that I should.*

(FG7, 39, Preschool teacher)

This quote suggests some of the female gamers considered engaging with the social gaming community more. One of the potential benefits of participants exploring these communities is that amongst heavy gamers, previous studies have found that gamers active in such communities are less likely to suffer from problematic gaming symptoms, such as depression, compared with gamers with less online social interaction [59]. In other words, gaming communities can act as a protective factor. Based on these findings, it is important to minimise any obstacles for female gamers to engage socially online, such as the general perception of female gaming and the risk of experiencing discrimination or harassment. A study by Vella et al. [32] highlights that although both males and females experience toxicity and performance pressure in online gaming communities, it is important to implement design strategies that enable female players, in particular, to engage in social interactions in a safe manner. McLean and Griffiths [30] highlight that female gamers experienced loneliness and anxiety because of harassment and lack of social support online. Emotionally sensitive and shy gamers gain more online friends than other gamers, indicating that online gaming communities can be a significant channel for overcoming offline social inhibitions and can thereby increase well-being [30]. 

Some participants mentioned changes to their gaming habits: “*I’ll probably try some new games that I previously assumed were out of my comfort zone because I know realise how confused I was, and how I was the only one stopping myself from playing them*” (FG12, 23, PhD student). In this quote, FG14 appears to hold herself back when trying other games that might be considered more difficult or skill-demanding than what she normally plays. Easing the entry for females into gaming holds the potential of several advantages beyond gameplay, such as increased likelihood of entering STEM fields and engaging in mixed-sex friendship groups [32]. Alternative suggestions were offered:

*I think it’s going to be a slow process for women to be completely accepted into gaming. But it’s getting there. And I think the first big step was the publishers making games about badass women. And the next step is for the audience to fully accept that, I think it’s moving. Slowly but surely.*

(FG6, 24, Influencer and Sponsorship Manager)

Supporting the statement by FG6 that women are gradually more accepted into gaming is a study by Choi et al. [60]. It found that the success behind the online communities was driven mainly by female members at the time. Tendencies and discourses appear to be changing, although more can be done to increase equality and opportunities between male and female gamers. Ideally, female gamers should be able to feel similar to what FG6 expresses here:

*I’m part of little cultures online that discuss those games and, mostly extends to how I consider [being] a gamer. I enjoy playing games and I’m proud about it, and I’ll tell everyone who wants to know, yeah I like games and I’m a woman. I’m not good at games, absolutely not, but I enjoy them and I guess that’s the most important thing.*

(FG6, 24, Influencer and Sponsorship Manager)

Accordingly, female gamers should be able to enjoy gaming and engage in social aspects of gaming, independent of their skill level, the type of game, and their gender.

## 4. Conclusions

This qualitative study presents an in-depth exploration of being a female gamer from participants who regularly play video games. Four themes were developed which best represented the participants’ experiences. These themes included the self-image of being a female gamer, the benefits and drawbacks associated with gaming, and finally ambitions and intentions. 

Findings indicate the difficulty of a self-image as female gamer. The stereotype of a male gamer is ever present despite gaming audiences being almost equally divided by gender. Gaming-related stigma persists and influences how female gamers perceived themselves. Strategies were used to avoid gender perception problems within games, such as the use of male avatars. This is consistent with previous research, where female gamer identity was related to perceived threat or stigmatization [61]. Researchers suggested that having a close relationship with other women discouraged gamer identity [61]. However, we found that experiences of stigma or discrimination from male gamers discouraged female gamers from labelling themselves as gamers or revealing their gender in-game. To combat such prejudices, Paaßen et al. [4] suggest that more women need to be visible in the role of gamer to create change. However, the first step appears to be that women need to accept they are an equal part of the gaming population [2,3]. From the side of the gaming industry, it is recommended to facilitate technical improvements so that women can game similarly as men in terms of respect, avoiding harassment, and sexual objectification [8]. 

Despite this negative self-image, immersion, social connection, and achievement were highlighted as the main benefits for female gamers, similar to those seen by male gamers [57]. The present study extends this as female gamers demonstrated additional gaming motivations. These were primarily engaging with the aesthetics or narratives of games. Gaming was often recognised as a positive experience across participants. However, comparable with male gamers, women also experienced levels of psychological distress and gaming-related mental health problems, including GD. However, GD appeared less common in our female sample than in male gamers [22]. This may be related to the ambivalence of female gamers playing particular games, such as MMORPGs and FPSs, which have been associated with an increased GD risk [62]. Gaming as a coping mechanism or escapism, as used by our participants, has been linked to excessive technology use and psychopathology [41]. This suggests that the mechanisms used by female gamers to support their mental health may have detrimental impacts. Therefore, more research is needed to identify risk and protective factors for mental health problems and GD within female gamers. 

Several participants discussed their gaming habits as hardest to control during their adolescence when their gaming may have been problematic. However, participants felt that they better managed these potentially problematic habits as they became older. Therefore, it is sensible to target young and middle-aged women with addiction prevention programmes and interventions. A previous qualitative study with a large female sample described gaming as an acquired, enjoyable habit starting in adolescence [62]. However, gaming was seen to progressively become more problematic for the user (e.g., loss of time, feelings, cognitions, relationships, activities) [62]. To counter this, women paid attention to their immersion gaming motivations. This reflects the gaming populations’ preference for prevention strategies rather than curative actions, favouring decision-making, autonomy, and self-directed actions [63]. Therefore, to better manage problematic gaming, interventions should focus on including the gamer and educating them to have greater awareness of their habits rather than enforcing system shut-down features and other modifications [63,64]. 

While the current study has many strengths, there are several limitations. First, participants were recruited following the completion of a previous study [22], which was posted on Reddit. Use of this site allowed free and rapid data collection [65]. However, this required targeted adverts in moderated subreddit threads, which were often separated by game type. This means we may have missed a sample of female gamers who primarily play one type of game. Second, while offering multiple interview methods remove graphical and financial barriers for participants, non-visual interviews, particularly written interviews, limit rapport building and the interpretation of nonverbal cues. 

In summary, female gamers make up half of the overall gaming population in West-ern countries, although they are underrepresented in the current literature [4]. The present study is one of few empirical studies investigating the construction, self-image and experiences of female gamers. It has shown that they have a history as gamers from adolescence, but still face internalised stigma. Externally, female gamer stigmatisation may result in sexism, gender violence, harassment, and objectification [8]. Additionally, females may decide against identifying as gamers and engaging in social gaming interaction, or they may hold back from online gaming in general. As a consequence, they may miss out on the opportunities for recreation as well as social and psychological benefits that gaming brings. There is, therefore, urgent need for more research and actions to promote change, equity, education, and security for female gamers.

## Figures and Tables

**Table 1:** Participant characteristics: age and occupation.

Participant	Age	Occupation
FG1	33	Researcher
FG2	27	PhD student
FG3	29	PhD student
FG4	35	Radiographer
FG5	28	Unemployed
FG6	24	Influencer and sponsorship manager
FG7	39	Preschool teacher
FG8	28	Unemployed
FG9	25	Marketing analyst
FG10	25	Software engineer
FG11	24	Data curator
FG12	25	PhD student
FG13	24	Research assistant (RA)
FG14	23	PhD student
FG15	21	Retail assistant
FG16	23	Lab technician
FG17	21	Donut vendor
FG18	27	PhD student
FG19	26	PhD student and RA
FG20	26	Localisation tester

## Data Availability

Data can be requested from the corresponding authors.

## Appendix A. Interview Questions

Female Gaming

Interview questions (Aiming: 1 h)

Explore the impact of gaming (both positive and negative) on females using qualitative methodologies.

Demographics:

- To skip some of these; what was your unique identifier in the survey?
- Could I just ask how old you are please?
- What are you currently doing for work? What are you enjoying outside of work?

Questions found in ethics

When I say “female gaming” what does this make you think about? What is important?

What kind of gaming do you do?

How do you experience your gaming?

- What meant this activity to you?
- Why do you game?
- What kind of utility does it serve for you? (Prompt: reduced negative emotions)
- Now, from a woman perspective, How do you perceive your gaming as a woman specifically? Do you think gaming is perceived similarly from men and women?
- How do you feel about the way women are portrayed in video games and gaming culture in general? (Prompt: Lara Croft effect, Perez vs Day Twitter incident)
- Do you think this has ever influenced the way you perceive yourself? For example, physical/psychological comparisons.
- How are women represented in the games? Please, could you help us to know about this ‘gender representation’? In other words, what type of women appear in the games? What do you think about them? Ideally, how would you like to see women presented in video games?
- Do you think that more women are starting to play games? Why? When? How do you think the way women are portrayed in video games influences sales?
- How and why do you think this is important?
- How do you perceive yourself as a gamer?
- To what extend do you think you identify as a gamer? (Prompt: part of the culture online, just offline gaming) and as a female gamer? Have you ever reflected on that? Why?
- Do you think this is different to how a man might consider himself as a gamer?
- Does this relate to the games which you play, how so?

What is different between female gaming and male gaming?

What kind of impact does gaming have on you, both positive and negative?

How do you positively experience your gaming?

How do you negatively experience your gaming?

About the negative, do you think ‘gaming addiction’ could affect females?

i. How?

ii. Why?

iii. How does this compare with males, what do you think?

What kinds of consequences has gaming had on you?

If you could change your gaming, what would you do?

What makes gaming important to you?

In research, there’s been some evidence which looks at a concept called game transfer phenomena. This looks at how immersed people can get into video game environments, which influences their thoughts, fantasies, and actions during or after play. This can more typically occur when game elements associate with real life elements, triggering thoughts, sensation or actions.

- Can you remember being so into a game that you noticed a change in thoughts, action or fantasies based on or around the game, and if so, how did these change?
  - (Prompt: For example, do you dream about the game or mix up details about a character and someone close to you?)
  - How did you feel during this experience?
- If you’ve had an experience of game transfer phenomena or something similar has this influenced your gaming, and if so, how?
- Alternatively, if you’ve had an experience of game transfer phenomena or something similar how has that effect your real-life behaviour, even just that one time?

Finally, a part of this phenomenon, because of your gaming, do you think you have ever experienced a problem? What type? What was the reason? Any other gamers involved or other persons in your life outside the game? Why this happened to you? When? How many times? What period? How did you cope with it?

Anything else to add that will help us to know more about female gamer and health issues?

Thank you for taking part, is there anything else you think that is important that I haven’t asked you?

Is there anything you’d like to ask me?
